# Epithelial Abnormalities in the Small Intestine of Zambian Children With Stunting

**DOI:** 10.3389/fmed.2022.849677

**Published:** 2022-03-16

**Authors:** Chola Mulenga, Sanja Sviben, Kanta Chandwe, Beatrice Amadi, Violet Kayamba, James A. J. Fitzpatrick, Victor Mudenda, Paul Kelly

**Affiliations:** ^1^Tropical Gastroenterology and Nutrition Group, University of Zambia School of Medicine, Lusaka, Zambia; ^2^Washington University Center for Cellular Imaging, Washington University School of Medicine, St. Louis, MO, United States; ^3^Departments of Cell Biology & Physiology and Neuroscience, Washington University School of Medicine, St. Louis, MO, United States; ^4^Department of Biomedical Engineering, Washington University in St. Louis, St. Louis, MO, United States; ^5^Department of Pathology and Microbiology, University of Zambia School of Medicine, Lusaka, Zambia; ^6^Blizard Institute, Barts & The London School of Medicine, Queen Mary University of London, London, United Kingdom

**Keywords:** environmental enteropathy, environmental enteric dysfunction (EED), microvilli, Africa, malnutrition

## Abstract

**Background:**

Environmental enteropathy (EE) contributes to impaired linear growth (stunting), in millions of children worldwide. We have previously reported that confocal laser endomicroscopy (CLE) shows fluorescein leaking from blood to gut lumen *in vivo* in adults and children with EE. We set out to identify epithelial lesions which might explain this phenomenon in Zambian children with stunting non-responsive to nutritional support.

**Methods:**

We performed confocal laser endomicroscopy (CLE) in 75 children and collected intestinal biopsies for histology in 91 children. CLE videos were evaluated, employing the Watson score to determine severity of leakiness. Morphometry was carried out on well-orientated mucosa and 3 biopsies were examined by electron microscopy.

**Results:**

Confocal laser endomicroscopy demonstrated substantial leakage from circulation to gut lumen in 73 (97%) children. Histology consistently showed characteristic changes of EE: villus blunting, lamina propria and epithelial inflammation, and depletion of secretory cells (Paneth cells and goblet cells). Epithelial abnormalities included marked variability in epithelial height, disorganised and shortened microvilli, dilated intercellular spaces, pseudostratification, formation of synechiae between epithelium on adjacent villi, crypt destruction, and abundant destructive lesions which may correspond to the microerosions identified on CLE.

**Conclusion:**

Epithelial abnormalities were almost universal in Zambian children with non-responsive stunting, including epithelial microerosions, cell-cell adhesion anomalies, and defects in secretory cells which may all contribute to impairment of mucosal barrier function and microbial translocation.

## Introduction

Stunting in young children is defined as length for age Z-score <-2.0 (1). Stunting is a pervasive problem in low and middle income countries (LMICs) worldwide (2), and is associated with increased mortality (3), impaired neurocognitive development (4) and blunted responses to oral vaccines (5, 6). A large body of evidence shows that nutritional supplementation does not overcome childhood stunting in LMICs (7), even when given during early pregnancy (8), possibly because of endemic environmental enteropathy (EE) in pregnant women (9, 10). It is not clear how much of the dysfunction is attributable to altered composition and function of the microbiome, but recent evidence that microbiota-directed complementary foods can improve growth (11) suggests that it may be a substantial contribution. EE is a chronic condition of the small intestine combining loss of villous architecture, reduced absorptive function, and intestinal and systemic inflammation. We have previously identified epithelial lesions as central features of enteropathy in children with severe acute malnutrition (SAM), where enteropathy drives microbial translocation and may correlate with adverse outcomes (12).

The spectrum of small intestinal disorders described as “enteropathy” has expanded recently. These enteropathies share many pathological features, and many of them lead to malnutrition and both mucosal and systemic inflammation. Coeliac disease was described by Gee in 1880, and tropical sprue was described by Manson in 1888, but older descriptions of clinical syndromes resembling these enteropathies date back centuries (13). Coeliac disease, like environmental enteropathy, may be manifest as malnutrition in childhood, through a combination of maldigestion, malabsorption, and inflammation. Infective enteropathy includes Whipple’s disease, non-tuberculous mycobacteriosis, strongyloidiasis, and other persisting infections. Persisting bacterial and protozoal infections are potent and highly prevalent causes of malnutrition in children (14). Immunological enteropathies, all rare, include graft-versus-host disease (GVHD), common variable immunodeficiency (CVID), CD55 deficiency (15), transplantation rejection enteropathy (16), and autoimmune enteropathy (17, 18). Two drug-induced enteropathies, olmesartan enteropathy (19) and immune checkpoint inhibitor enteropathy (20) are probably also immune-mediated. Radiation-induced small intestinal enteropathy is induced by DNA damage in stem cells and damage to vascular endothelium (21). The key role of prostaglandins in maintaining small intestinal mucosal integrity is emphasised by the role of non-steroidal anti-inflammatory drugs (NSAIDs) (19) and SLCO2A1 mutations (22) in inducing enteropathy. These enteropathies all share villus blunting and inflammation as prominent pathological abnormalities, and it is likely that reduced surface area plays a major role in driving the impaired nutritional state which is a common clinical manifestation.

Recent work has achieved a degree of consensus among pathologists on key histological features of environmental enteropathy (23). Villus architectural remodelling (villus blunting) is a key feature of enteropathy, as was described decades ago (24, 25). Loss of goblet cells and Paneth cells is also a prominent feature of EE, in common with autoimmune enteropathies (17). Epithelial thinning, also referred to as flattening, or cuboidal change, is also a prominent feature of autoimmune enteropathies (17). We recently reported leakage from circulation to gut lumen in adults (26) and children (27) with EE using confocal laser endomicroscopy (CLE). Here, we assess abnormalities of the epithelium in children with environmental enteropathy to identify epithelial pathology that might explain leakiness of the small intestinal mucosa, combining CLE, histology, and electron microscopy.

## Materials and Methods

The BEECH study has been described in detail previously (27). In brief, children under 12 months of age in Misisi, Lusaka, were screened for evidence of growth failure. Those with weight-for-age *z* (WAZ) score of ≤-1 were invited to come to the local research clinic, St Augustine Clinic, for further evaluation. Those with length-for-age z (LAZ) scores of –2 or less were offered daily nutritional supplementation with corn-soy blend (28) (1.5 kg/week), an egg, and a single micronutrient sprinkle (Nutromix, Hexagon Ltd., Mumbai, India), for periods of 4–8 months. Those with no evidence of response (consistent LAZ score of ≤–2 over at least 1 month) were investigated further (29), including upper gastrointestinal endoscopy, biopsy for morphometry and confocal laser endomicroscopy (27). Ethics approval for this study was obtained from the University of Zambia Biomedical Research Ethics Committee (approval 006-02-16 dated 31 May 2016), and written consent was obtained from all the parents and guardians of children participating in the study.

We performed endoscopy with a Pentax EG-2490k gastroscope with external diameter 8mm and working channel diameter of 2.4 mm. CLE was performed before biopsies were collected, using the Cellvizio system (Mauna Kea Technologies, Paris, France) with Alveoflex probes as previously reported (27). After obtaining a stable viewing position in the second part of the duodenum, and avoiding trauma to the mucosa, 2 mL of 1% fluorescein was injected intravenously and images were collected continuously for one minute. Videos were evaluated by a single observer (VK); after initial fogging and excluding periods of non-contact or movement artefact the median duration of evaluable imaging was 50 (IQR 39–56, range 27–106) s. The Watson score (30) was used to evaluate the severity of epithelial leakiness, but as the Alveoflex has lower resolution than the Pentax EG3870CIK confocal laser endomicroscope we used previously (26) it was difficult reliably to discriminate between Watson grades 2 and 3 which requires reliable identification of microerosions.

Biopsies were collected into normal (0.9%) saline and orientated under a dissecting microscope, then placed on cellulose acetate paper (Sartorius GmbH, Gottingen, Germany) in neutral buffered formal-saline (CellPath Ltd., Newtown, Powys, United Kingdom), and processed into wax blocks. Sections of 3–4 μm were stained with haematoxylin and eosin and then scanned on an Olympus VS120 slide scanning microscope at 20× magnification, as previously described (27), in order to generate measurements of villus height (VH), crypt depth (CD), and epithelial perimeter in relation to length of muscularis mucosae (Supplementary Figure 1). All slides were reviewed for features which could contribute to an understanding of epithelial leakiness. Morphometry was carried out on well-orientated mucosa (Supplementary Figure 1). The criterion for orientation of a section or part of a section was that each crypt should be seen throughout its length. Where this criterion was satisfied, all villus and crypt units, the overlying epithelium, and the underlying muscularis mucosae, were measured. Especial care was taken to include small villus units which represent the beginning and end of a ridge-like villus. Epithelial and brush border heights were also measured in the same sections. One biopsy each from three participating children selected at random were placed without orientation into glutaraldehyde buffer (Sigma, Poole, United Kingdom) for electron microscopy and transported to the Washington University Center for Cellular Imaging (St. Louis, MO, United States) where they were further rinsed in 0.15 M cacodylate buffer with 2 mM calcium chloride three times for 10 min each and subjected to a secondary fixation for 1 h in 1% osmium tetroxide/1.5% potassium ferrocyanide in cacodylate buffer. Following this, samples were rinsed in ultrapure water three times for 10 min each and incubated in an aqueous solution of 2% uranyl acetate overnight at 4°C. The samples were then rinsed in ultrapure water 3 times for 10 minutes each, dehydrated in a graded ethanol series (30%, 50%, 70%, 90%, 100% 3x) for 10 min in each step, and infiltrated with microwave assistance (Pelco BioWave Pro, Redding, CA, United States) into Epon resin. Samples were embedded and cured in an oven at 60°C for 72 h. Post resin curing, samples were sectioned and 70 nm thick sections were stained with aqueous 2% uranyl acetate and Reynold’s lead prior to imaging on a transmission electron microscope (TEM JEOL JEM-1400 Plus) at 120 kV.

## Results

**FIGURE 1:**
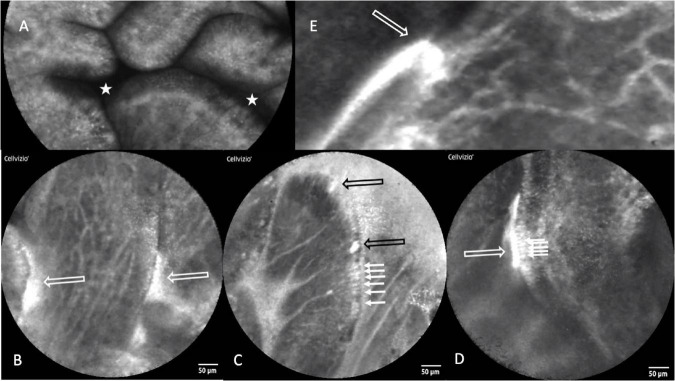
Confocal laser endomicroscopy images of environmental enteropathy in Zambian children with stunting. **(A)** Normal image showing fluorescein, which was injected intravenously, in villus epithelium and lamina propria and no signal in the lumen which therefore appears dark (star). **(B)** Fluorescein leakage from epithelium as plumes (white hollow arrows). **(C)** Fluorescein highlighting intercellular spaces (small arrows) and two microerosions (black hollow arrows). **(D)** Fluorescein highlighting intercellular spaces and leaking into gut lumen (small arrows). **(E)** Fluorescein plume (white hollow arrow) originating from capillaries, the escaping through defect in epithelium and into lumen.

Intestinal biopsies of adequate quality for assessment were obtained from 91 children with stunting, and CLE videos were obtained in 75 procedures [Table 1 and reference (27)]. Biopsies from 27 children were unsatisfactory for mucosal evaluation. The images show consistent evidence of epithelial leakiness, demonstrated by plumes of fluorescein observed flowing from systemic circulation, into which the fluorescein was injected, into intestinal lumen (Figure 1). Images from 73 (97%) children had Watson scores of 2 or 3 and only two (2.7%) had a score of 1. CLE showed fluorescein in intercellular spaces between epithelial cells (Figure 1) in 46 (62%) of videos, although this was usually patchy and variable in extent. Occasional microerosions were also recorded (Figure 1D). Light microscopy revealed a pathogen (*Giardia intestinalis*) in one biopsy, which had not been detected on stool examination.

**TABLE 1:** Clinical and demographic characteristics of children who had biopsies and the subset who had confocal laser endomicroscopy.

	Biopsy set (*n* = 91)	CLE set (*n* = 75)
Sex (M:F)	45:46	40:35
Age (months)	18 (15,21)	18 (15,21)
LAZ	–3.27 (–3.91, –2.7)	–3.29 (–3.91, –2.89)
WLZ	–0.68 (–1.34, –0.21)	–0.61 (–1.29, –0.13)
WAZ	–2.25 (–2.68, –1.77)	–2.27 (–2.68, –1.85)
**HIV status**		
Infected	1	1
Unexposed	60	56
Exposed, uninfected	29	17

*Values shown are median (IQR).*

**FIGURE 2:**
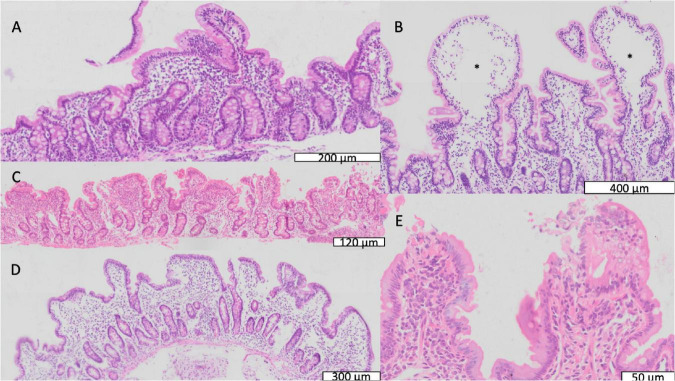
Mucosal features of environmental enteropathy. **(A)** Marked villus blunting in a well-orientated section (confirmed by crypts imaged in full longitudinal section). **(B)** Ballooning of villus tips caused by expansion of translucent material or oedema marked by *. **(C)** Pronounced inflammatory infiltrate in lamina propria. **(D)** Extensive villus fusion, in which fused villus structures overlie several crypts. **(E)** Loss of epithelial integrity at villus tips representing microerosions.

Biopsies showed a wide range of appearances, from mild inflammation to severe villus atrophy (Figure 2 and Table 2). On the whole VH was reduced compared to a UK reference range (Table 2), and 80 (88%) of 91 children had villus blunting, defined as below the United Kingdom reference range. Lymphoid aggregates and penetration of the mucosa by Brunner’s glands were also observed (Supplementary Figures 2, 3). Three of the biopsies demonstrated ballooning of the villi due to expansion of the lamina propria (Figure 2).

**TABLE 2:** Mucosal morphometry in biopsies from 91 stunted children.

Parameter	Values (median, IQR) [range]	Reference range
Villus height (μm)	184 (144,218) [65–435]	258–436 (mean 342, SD 42)
Crypt depth (μm)	171 (147,203) [110–415]	121–219 (mean 170, SD 25)
Epithelial perimeter (μm^2^)	530 (412,598) [216–1233]	
**Epithelial height (μ m)**		
Maximum	36.1 (30.0,47.1) [18.0–104.1]	
Minimum	12.3 (8.9,14.8) [4.0–22.7]	
Brush border height (μm)	1.36 (1.05,1.72) [0.55–3.99]	
BB below 1.3μm	42 of 91 (46%)	

*IQR, interquartile range; SD, standard deviation. Reference range was obtained from United Kingdom children [references (31, 32)] and their distributions merged using an online tool (http://www.obg.cuhk.edu.hk/ResearchSupport/StatTools/CombineMeansSDs_Pgm.php, accessed 29^th^ October 2021) then defined as mean ± 2SD. Villus height and crypt depth measurements have been published previously (27).*

### Intercellular Spaces and Junctions

**FIGURE 3:**
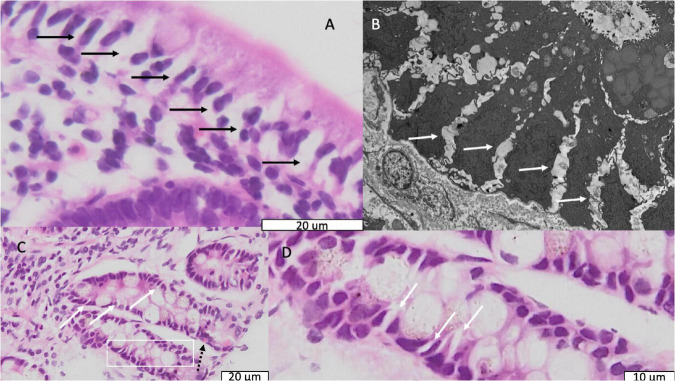
Light and electron micrographs showing dilated intercellular spaces. **(A)** Light micrograph of epithelium in a biopsy from a child with stunting; black arrows indicate dilated intercellular spaces. **(B)** Electron micrograph from the same child as panel **(B)**. Solid white arrows indicate dilated intercellular spaces. **(C)** Light micrograph of crypts in a biopsy from a different child showing the same dilated intercellular spaces; the dotted black arrow indicates an enteroendocrine cell. **(D)** Further magnification of the are in panel **(C)** represented by the white box showing the dilated intercellular spaces more clearly.

In 89 biopsies from 91 children the epithelium contained expanded intercellular spaces, and in 18 of these it was severe (Figure 3); this appeared consistent with the intercellular fluorescein accumulation seen in CLE videos (Figures 1D,E). This was corroborated by electron microscopy which demonstrated wide gaps in between epithelial cells (Figure 3).

### Epithelial Irregularities

**FIGURE 4:**
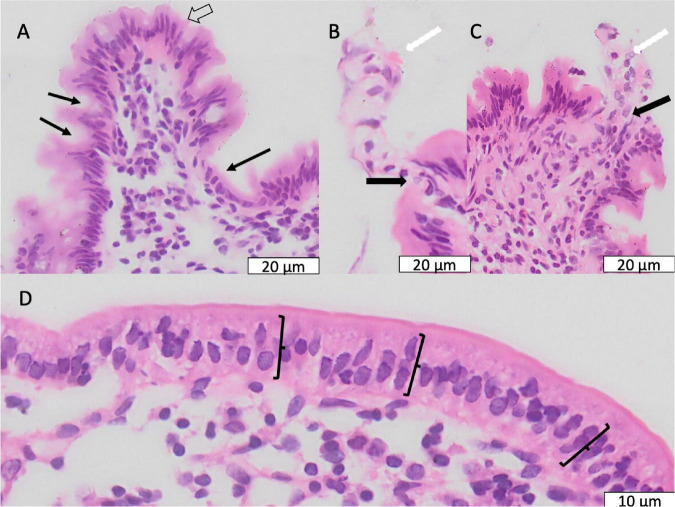
Enterocyte morphological abnormalities. **(A)** Variable epithelial height, ranging from full epithelial height to severe flattening (black arrows); at the villus tip there is also scalloping (white hollow arrow) which frequently resembled tufting. **(B,C)** Villus tip epithelial defect (solid black arrow) allowing extrusion of lamina propria contents (white arrows) into lumen. **(D)** Pseudostratification, the misalignment of nuclei into layers (range illustrated by brackets) which normally occupy a uniformly basal position in the enterocyte.

Epithelial height displayed variation between biopsies. There was also marked irregularity in epithelial cell height (Figure 4 and Table 2), which in some cases resembled tufting (Figure 4C). Pseudostratification was also observed (Figure 4D). Destructive lesions and microerosions which appear consistent with epithelial defects seen on CLE (Figure 1D).

### Secretory Cells

**FIGURE 5:**
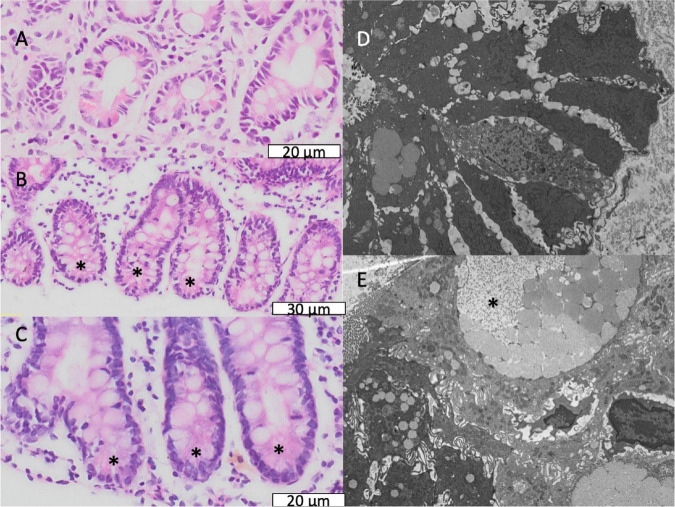
Secretory cell abnormalities in environmental enteropathy. **(A–C)** Paneth cell depletion; the expected location of Paneth cells is shown, normally recognisable by brightly eosinophilic granules. **(D,E)** Electron micrographs of goblet cells with depleted granules.

Characteristic features of environmental enteropathy were observed, including goblet cell depletion, Paneth cell degranulation, and intra-epithelial lymphocytosis (23) (Figure 5). Enteroendocrine cells (Figure 5) were seen occasionally. In contrast, healthy biopsies from individuals without EE show plump eosinophilic Paneth cell granules and abundant goblet cells (Supplementary Figure 4).

### Brush Border

**FIGURE 6:**
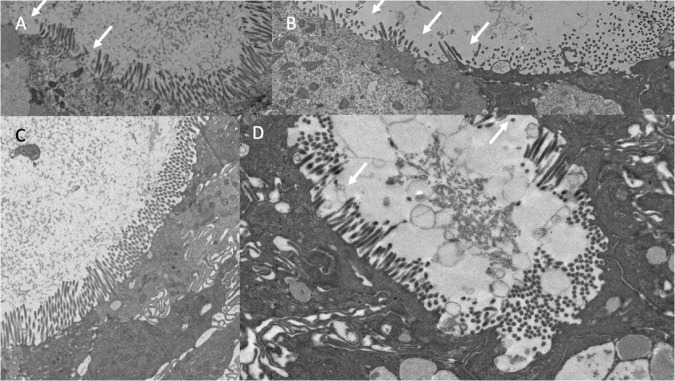
Brush border disruption in environmental enteropathy, ranging from minor loss of uniformity of microvilli **(A,C)** to marked loss of microvilli **(B,D)**.

The microvillus brush border (BB) was markedly irregular (Table 2 and Figure 6). Using a cut-off of 1.3μm to define shortened microvilli (33), 42 (46%) of biopsies showed BB thinning. VH correlated with BB (ρ = 0.24; *P* = 0.02) and epithelial height (ρ = 0.24; *P* = 0.02). Electron micrographs confirmed this irregularity of the brush border (Figure 6) but no pathogens were identified in sections imaged by EM as potential explanations for BB damage.

### Less Common Lesions

**FIGURE 7:**
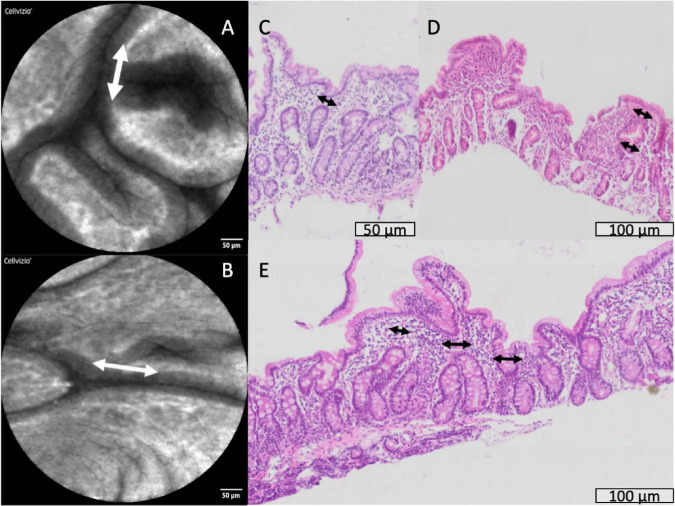
Synechiae (epithelial bridges, marked with arrows) progressing to villus fusion. These are shown by confocal laser endomicroscopy **(A,B)** and histology **(C–E)**.

In a small number of biopsies, severe lamina propria inflammation was associated with crypt architectural abnormalities (Supplementary Figure 5) or loss of crypts (Supplementary Figure 6). Synechiae were observed in CLE images and corresponding structures were observed in histological sections (Figure 7), closely mirroring previously reported synechiae in a range of enteropathies (34).

## Discussion

Epithelial leakiness in environmental enteropathy is a central pathophysiological feature, associated with microbial translocation and previously visualised *in vivo* in adults and children using confocal laser endomicroscopy (26, 27). Here we report visual evidence of epithelial damage which appears to underlie this leakiness, and which is also likely to be a feature of several enteropathic conditions. The remarkable feature of the severe abnormalities described in this paper is that these biopsies were taken from ambulant, stunted children with no specific symptoms. We have identified dilated intercellular spaces, epithelial irregularity (with pseudostratification and occasional similarities to tufting enteropathy), brush border damage, and loss of secretory cells in the epithelium. We also identified crypt destruction in some biopsies, and we speculate that this may help explain the reduction in proliferating cells we have observed in adults using single cell transcriptomic analysis (35). Epithelial flattening has long been recognised as a feature of coeliac disease (36), and variable flattening of epithelial cells was clearly evident in this study of environmental enteropathy, closely resembling the epithelial flattening observed long ago in “PEM” (protein energy malnutrition; an outmoded term which describes syndromes now called “Severe Acute Malnutrition”)(37). Pseudostratification (Figure 4D) was observed in a large proportion of biopsies, contrasting clearly with normal biopsies which show nuclei in an orderly basal position in the epithelial cell (38). Both epithelial flattening and pseudostratification are features of coeliac disease, and synechiae are visible in biopsies from coeliac disease (39). The significance of these morphological changes is uncertain, but pseudostratification is a prominent feature of intestinal morphogenesis during fetal, or foetal development (40, 41). It may therefore represent a more proliferative epithelium, or possibly dedifferentiation. The principal limitation of this study is the lack of normal biopsies from children without EE. It is difficult to justify obtaining normal tissue from healthy children, and healthy controls from the same population may also have degrees of enteropathy given the high carriage rates of potentially pathogenic microbes. Thus identifying suitable controls is difficult, and it may be that outlier groups from temperate countries may be needed. Anyway, it would be highly desirable to have control tissue to conclude definitely that the epithelial abnormalities reported here are exclusive to environmental enteropathy.

Confocal laser endomicroscopy in young children is inevitably constrained by the lower resolution of the endoscopes than we can achieve in adults, because of size limits on instruments that can be used. In adults we used a Pentax EG3870CIK confocal laser endomicroscope, which has a resolution of 1 μm but an external diameter of 18mm which is too large for use in young children. In the children reported here and in our previous publication, we used Alveoflex probes which have lower resolution, insufficient to image cellular detail consistently, but the visualisation of the leak of fluorescein is still very clear and reliable and some cellular imaging is possible. Consequently, we could not discriminate reliably between Watson grades 2 and 3 in all cases. Our data do, however, show clearly that young children with stunting in Zambia have surprisingly severe environmental enteropathy with pronounced epithelial leakiness. Only 2 out of 75 children had Watson scores of 1, which contrasts sharply with grade 1 scores in 80% of German adults sampled from the same part of the duodenum (42). We are not aware of similar interrogations of the small bowel in children in industrialised countries using CLE.

Many of the key findings in this study recapitulate earlier studies using electron microscopy in children with PEM (37) or marasmus (43). Given the severity of the clinical malnutrition in those earlier studies, which would now be termed severe acute malnutrition (SAM), this is surprising as the children reported in the present study were all ambulant and apparently healthy, though significantly stunted. Children with SAM in the earlier studies were all hospitalised. Both Brunser (43) and Shiner (37) reported reduced size and morphological abnormalities in the brush border, and Shiner’s report (37) mentioned reduced epithelial height which improved on nutritional rehabilitation. In our samples there was a great deal of variability in epithelial height, which often showed marked irregularity resembling tufting enteropathy. Tufting enteropathy is a severe congenital childhood enteropathy which has now been reproduced *in vitro* in an EpCAM mutation model (44). It would be of interest to determine if disturbances of EpCAM expression play a role in environmental enteropathy. There are some important features of EE which help distinguish it from, for example, autoimmune enteropathy (AIE), which shares loss of goblet and Paneth cells but does not display increased densities of IELs (45).

The physiological consequences of dilated intercellular spaces, if any, remain uncertain and any relationship to tight junction malfunction needs to be clarified. Paracellular flow through dilated intercellular spaces may permit passive transexudation of plasma proteins and other plasma constituents, and is likely to contribute to permeation of molecules such as lactulose (46–48). It may also allow for passive uptake of malabsorbed nutrients [e.g., zinc (49)] following oral administration, in the immediate post-prandial phase when luminal concentrations are higher than plasma concentrations.

The disruption of the brush border observed in our study would be expected to have consequences for digestion and absorption. We have previously reported reduced gene expression of a range of digestive enzymes including enterokinase, aminopeptidases A and N, two trypsinogens, diprolyl peptidase 4, folate hydrolase, angiotensin converting enzyme, alkaline phosphatase, maltase-glucoamylase, sucrose-isomaltase, lactase, and trehalase in children with malnutrition and environmental enteropathy (50). A range of nutrient transporters (SLC genes) were also reduced (50). Taken together with the evidence of brush border damage reported here, and older evidence of the link between brush border health and disaccharide malabsorption (51), this strongly suggests that damage to the microvilli contributes to malabsorption which may underlie environmental enteric dysfunction (9, 10, 52–55).

Depletion of secretory cells appears to be a consistent feature of EE (23). As goblet cells secrete the mucus layer, and Paneth cells synthesise a variety of antimicrobial peptides into it (56), such defects would be expected to lead to impaired barrier function. It seems likely that impaired secretory function is responsible for the intense microbial translocation which we and others have reported in EE (12, 27). The mechanism of secretory cell depletion is not understood, but it was not observed in experimental riboflavin deficiency in animals (57). Whether other specific nutritional deficiencies or pathobiont colonisation (11, 27) are responsible for the epithelial damage in EE is not clear, but a clear understanding of mechanism is clearly urgently needed.

## Data Availability Statement

The original contributions presented in the study are included in the article/Supplementary Material, further inquiries can be directed to the corresponding author.

## Ethics Statement

The studies involving human participants were reviewed and approved by University of Zambia Biomedical Research Ethics Committee. Written informed consent to participate in this study was provided by the participants’ legal guardian/next of kin.

## Author Contributions

CM, JF, VM and PK: study conceptualisation. CM, KC, BA, and PK: sample collection and processing. VM: biopsy scoring. VK: video scoring. SS and JF: electron microscopy. PK: first draft of the manuscript. All authors contributed to the manuscript.

## Conflict of Interest

The authors declare that the research was conducted in the absence of any commercial or financial relationships that could be construed as a potential conflict of interest.

## Publisher’s Note

All claims expressed in this article are solely those of the authors and do not necessarily represent those of their affiliated organizations, or those of the publisher, the editors and the reviewers. Any product that may be evaluated in this article, or claim that may be made by its manufacturer, is not guaranteed or endorsed by the publisher.
