# Supplementary material for: Epithelial Abnormalities in the Small Intestine of Zambian Children With Stunting
Source: Front Med (Lausanne). 2022 Mar 16;9:849677. doi: 10.3389/fmed.2022.849677 (PMC8966729; doi:10.3389/fmed.2022.849677)

**Epithelial abnormalities in the small intestine of Zambian children with stunting**

*Supplementary figures*

**Figure S1** Screenshot of morphometry performed on an Olympus VS-120 scanning microscope. Good orientation of the tissue allows 15 villus-crypt units to be measured for villus height (black), crypt depth (yellow), epithelial villus perimeter (white), and muscularis mucosae (green). To derive an estimate of epithelial surface area, the sum of epithelial perimeter is divided by the sum of muscularis mucosae length over which it was measured.


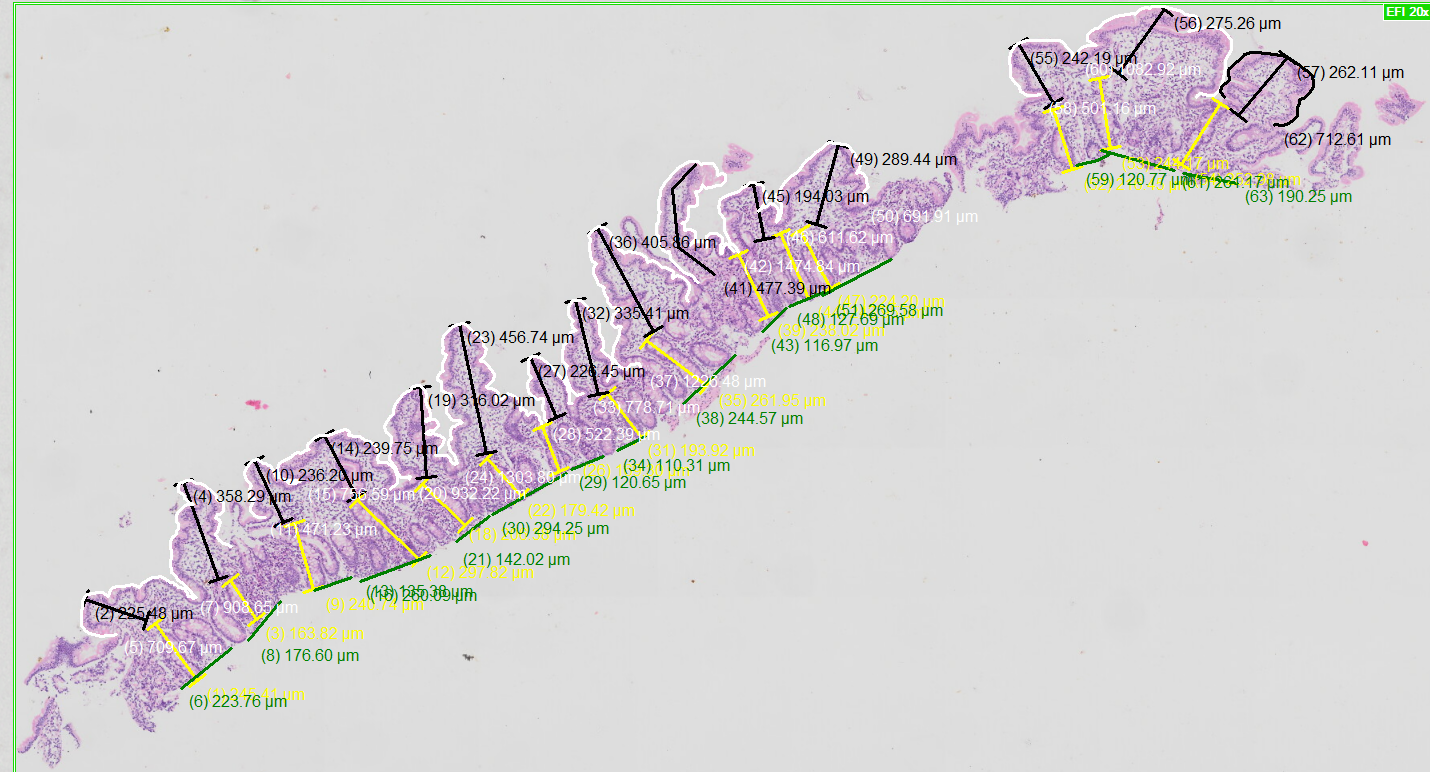


**Figure S2** Two images of lymphoid aggregates, an uncommon feature of EE


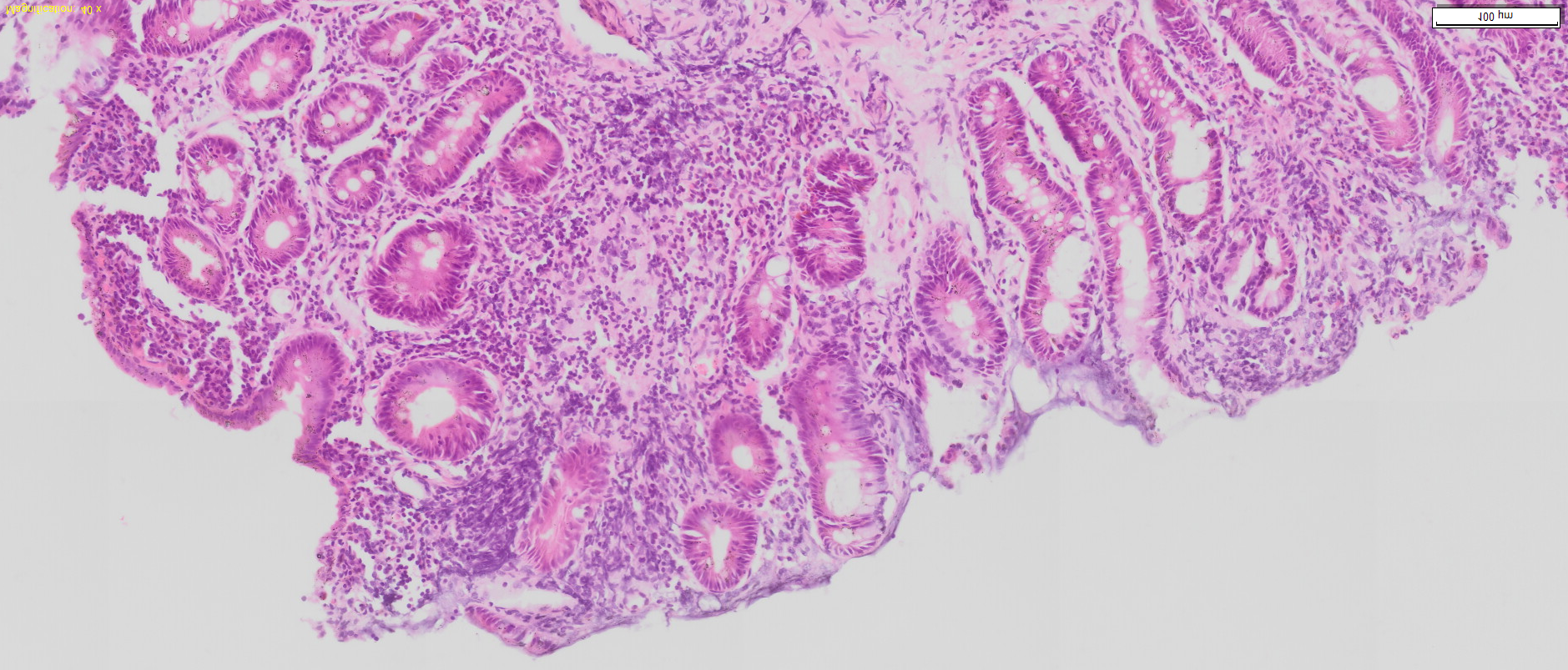


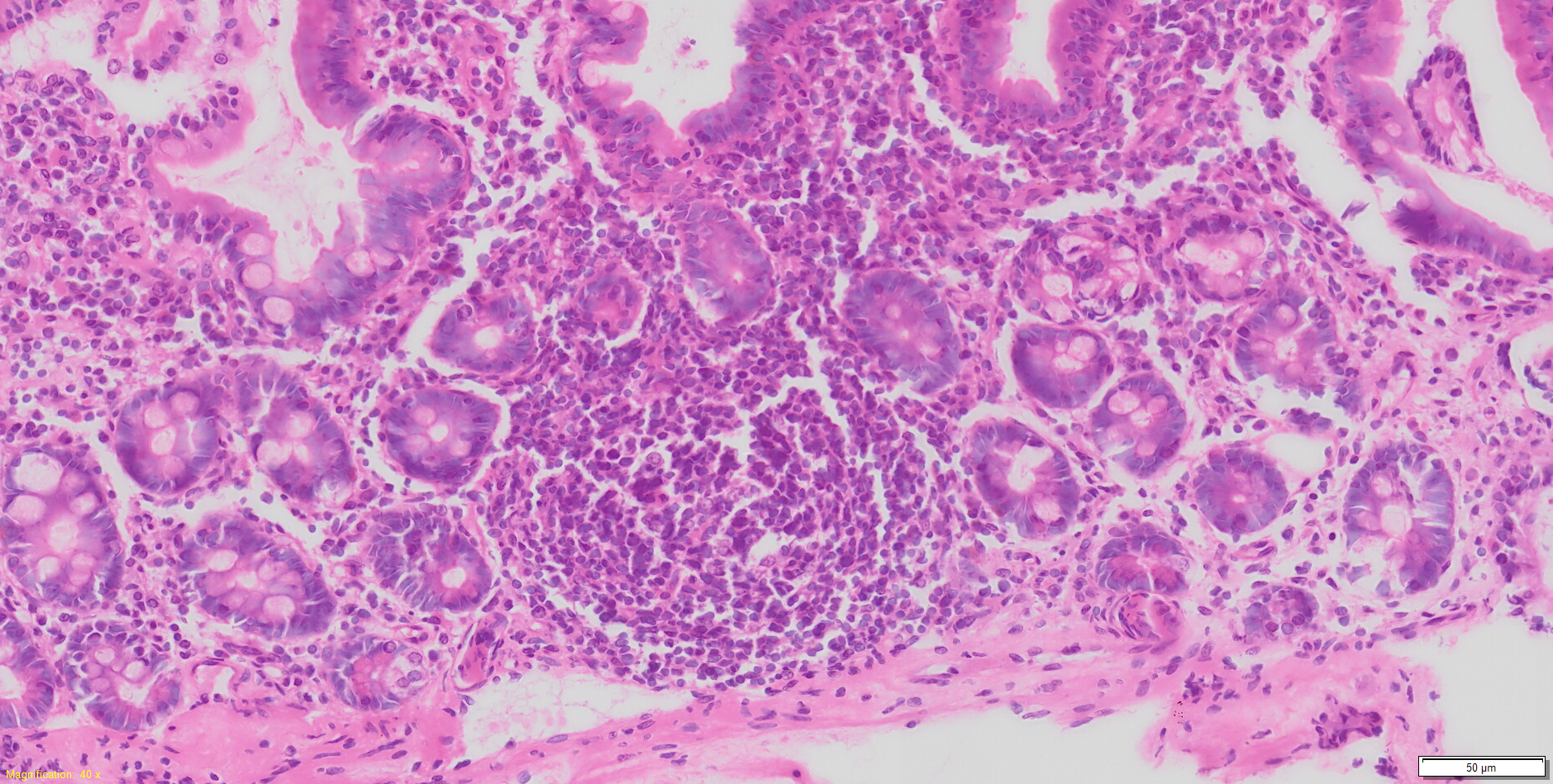


**Figure S3** Intramucosal Brunner’s gland penetration was an infrequent feature of EE in this cohort.


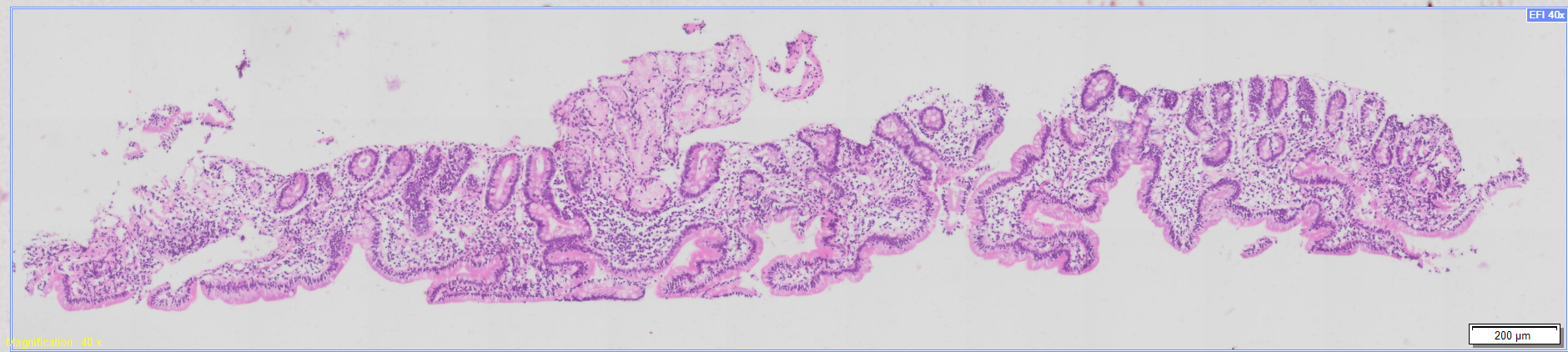


**Figure S4** Biopsies from duodenum unaffected by EE (courtesy of Ms Chola Mulenga), showing (A) Paneth cells with plump eosinophilic granules (arrow), and (B) lower magnification overview showing abundant goblet cells (see arrow indicating example goblet cells which have clear granules).

**Figure S5** Two rare examples of crypt architectural distortion, showing branched crypts (arrows) which are generally believed to indicate long-standing inflammatory processes and can be observed in ulcerative colitis.


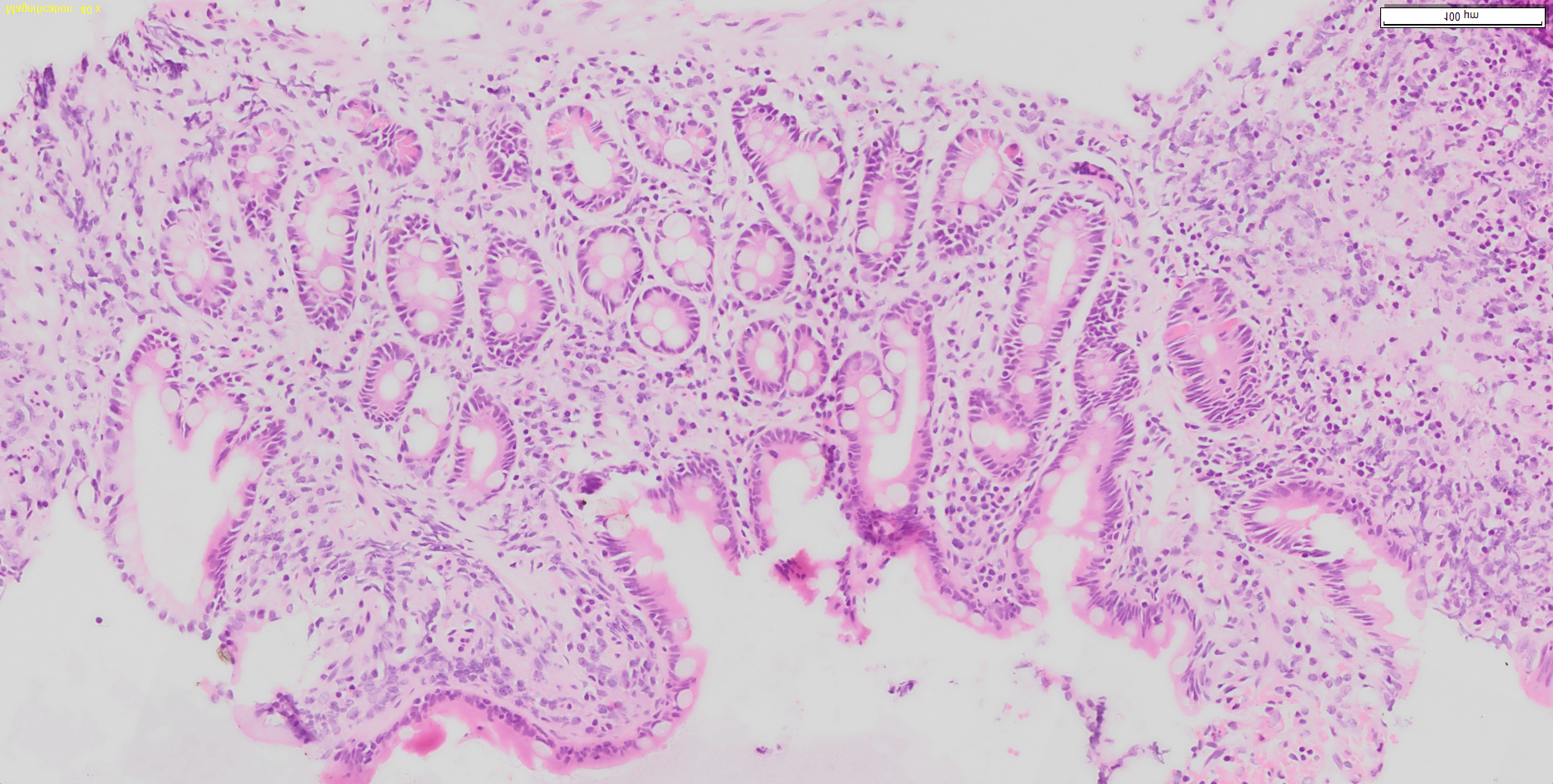


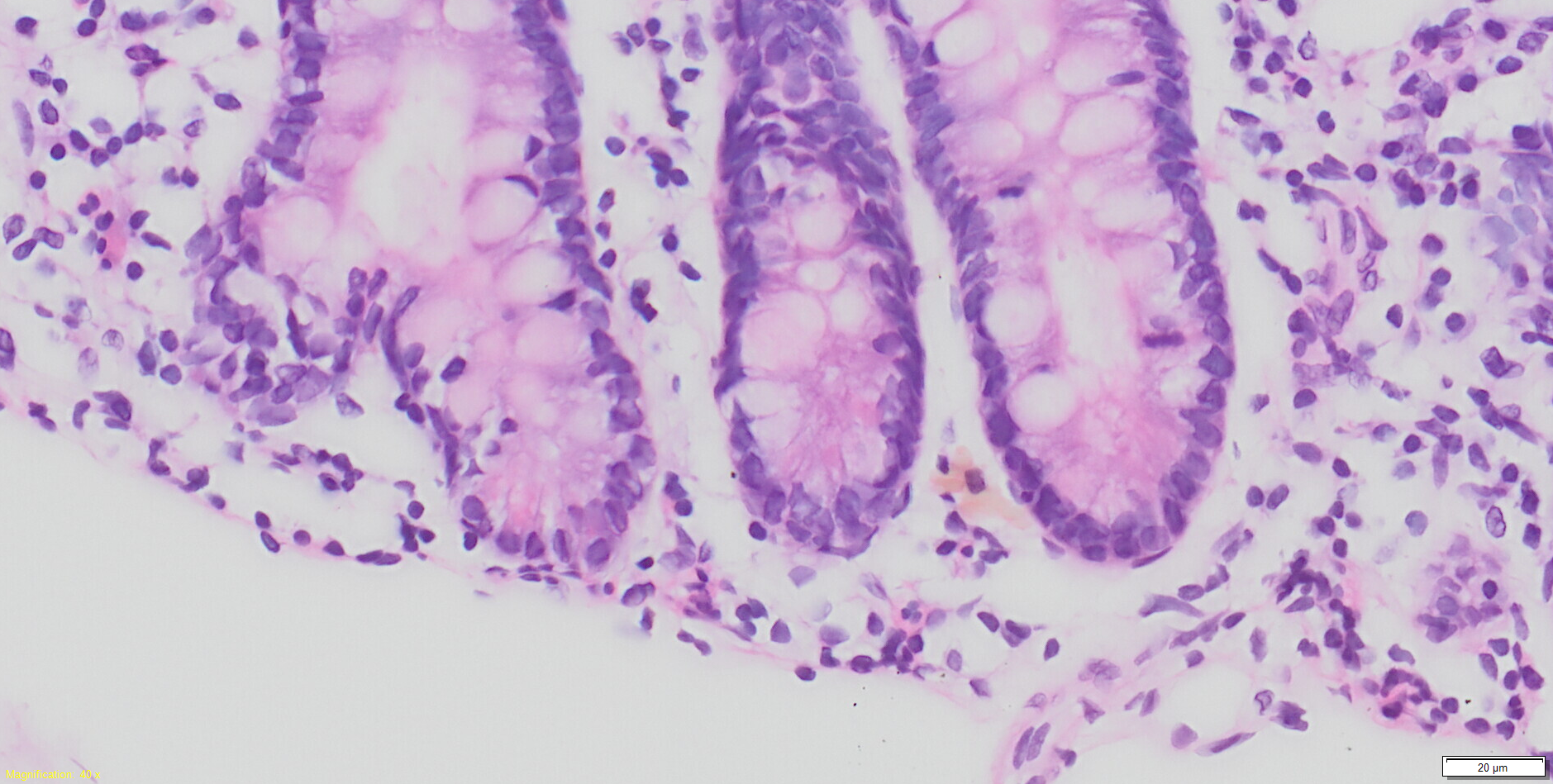


**Figure S6** Crypt destruction in several examples of biopsies characterised by severe lamina propria inflammation. Crypt ‘ghosts’ can be seen in panels B, C, and D, while in panels A and B crypts have been replaced by inflammatory aggregates.


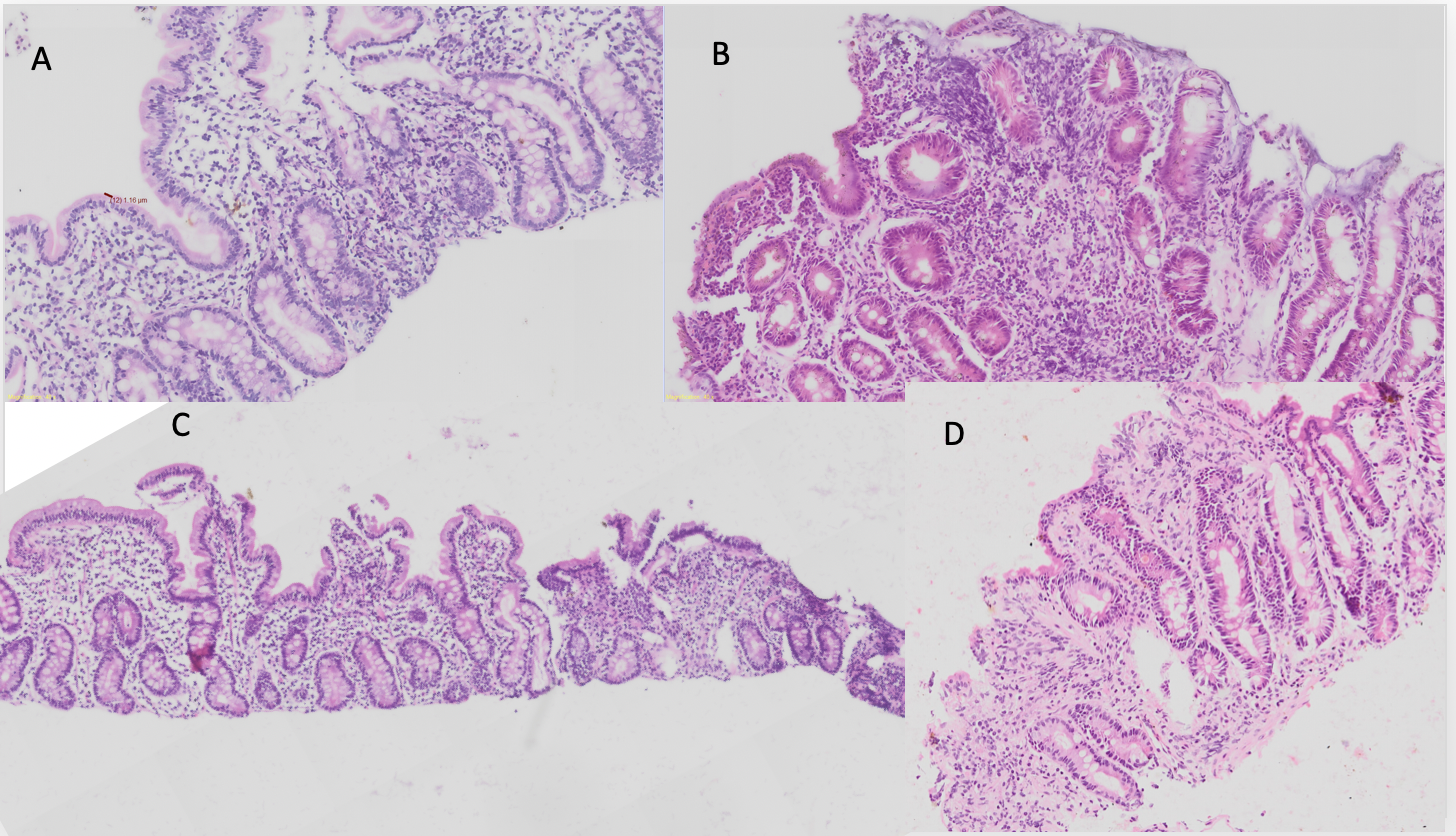

Supplement: Supplementary file 1 [file Data_Sheet_1.docx]
